# Sight restoration after congenital blindness does not reinstate alpha oscillatory activity in humans

**DOI:** 10.1038/srep24683

**Published:** 2016-04-15

**Authors:** Davide Bottari, Nikolaus F. Troje, Pia Ley, Marlene Hense, Ramesh Kekunnaya, Brigitte Röder

**Affiliations:** 1 Biological Psychology and Neuropsychology, University of Hamburg, Von-Melle-Park 11 20146 Hamburg, Germany; 2 Department of Psychology, Queen’s University, 62 Arch Street, K7L 3N6 Kingston, Ontario, Canada; 3 Canadian Institute for Advanced Research, 180 Dundas Street West, Suite 1400, M5G 1Z8 Toronto, Ontario, Canada; 4 Jasti V Ramanamma Children’s Eye Care Center, LV Prasad Eye Institute, Kallam Anji Reddy Campus, L V Prasad Marg, Banjara Hills, 500 034 Hyderabad, Andhra Pradesh, India

## Abstract

Functional brain development is characterized by sensitive periods during which experience must be available to allow for the full development of neural circuits and associated behavior. Yet, only few neural markers of sensitive period plasticity in humans are known. Here we employed electroencephalographic recordings in a unique sample of twelve humans who had been blind from birth and regained sight through cataract surgery between four months and 16 years of age. Two additional control groups were tested: a group of visually impaired individuals without a history of total congenital blindness and a group of typically sighted individuals. The EEG was recorded while participants performed a visual discrimination task involving intact and scrambled biological motion stimuli. Posterior alpha and theta oscillations were evaluated. The three groups showed indistinguishable behavioral performance and in all groups evoked theta activity varied with biological motion processing. By contrast, alpha oscillatory activity was significantly reduced only in individuals with a history of congenital cataracts. These data document on the one hand brain mechanisms of functional recovery (related to theta oscillations) and on the other hand, for the first time, a sensitive period for the development of alpha oscillatory activity in humans.

The posterior alpha oscillatory activity (8–12 Hz) of the human electroencephalogram (EEG) has been associated with the inhibition of task-irrelevant neural circuits^1^ and has been considered as a mechanism for coordinating neural activity in a large number of perceptual and cognitive tasks^2^. Alpha activity increases (alpha synchronization) result in an inhibition of task-irrelevant neural circuits, alpha decreases (alpha desynchronization) are associated within an engagement of task-relevant neural populations^1^,^2^. Moreover, the level of pre-stimulus alpha was found to predict visual processing efficiency^3^,^4^ and seems to be important for the control of attention^5^. Alpha oscillations dominate in the granular and infragranular layers of the cortex; they most likely result from the activity of pyramidal cells modulated by pulsed GABA (Gamma-amino-butyric acid) mediated inhibition of fast-spiking inhibitory interneurons^1^,^6^.

The EEG of a newborn is characterized by lower frequency activity including theta oscillations^7^. Posterior alpha activity seems to hardly exist in the EEG of newborns^7^ and shows a protracted developmental time course lasting until late childhood^8^. Posterior alpha activity during rest^9^,^10^ and in somatosensory tasks^11^,^12^ has been found to be markedly reduced or even absent in humans who had been totally blind since birth or who had experienced no more than unstructured light sensations. In contrast, oscillatory activity in lower (e.g. delta) and higher frequency bands (e.g. gamma) during rest have been reported to be relatively unimpaired in individuals with permanent congenital blindness^13^. Interestingly, people who had lost their sight as adults showed a gradual decrease of alpha activity over the following months^11^. These findings suggest that alpha oscillations crucially depend on structured visual input. However, it is not yet known, whether the emergence of the neural mechanisms generating alpha oscillations is linked to a sensitive period during which visual experience must be available. Individuals who had their sight restored after a congenital total blindness offer the unique possibility to address this question. In fact, individuals who were born with dense bilateral cataracts which were removed later in life provide such a model in humans^14^,^15^,^16^ and allow uncovering sensitive periods analogous to the extensive work in animals^17^,^18^.Strong evidence in favor of a sensitive period for the emergence of the neural mechanisms underlying alpha oscillatory activity would be if it could be shown that alpha oscillations do not or not fully recover after restoring sight following a congenital blindness while alpha oscillations would be recorded in visually impaired individuals who had had some vision at birth.

Here we recorded the electroencephalogram (EEG) from twelve individuals who had been born blind due to dense bilateral congenital cataracts (cc) while they were processing intact and scrambled biological motion stimuli. Recent evidence has suggested that the behavioral sensitivity to biological motion is indistinguishable for congenital cataract-reversal individuals and matched controls^19^,^20^. Thus, investigating oscillatory activtiy in the context of biological motion processing allows us to identify the neural mechanisms of functional recovery. Moreover, by studying alpha activity we were able to assess neural mechanisms related to the control of the excitatory/inhibitory balance of neural ciricuits. Since the latter crucuially rely on GABA-mediated mechanisms, we consider alpha oscillatory activity as an index for the functionality of inhbitory circuits whose establishment has often been postulated to constitute a hallmark of brain development^21^.

In order to control for the impact of factors related to cataract surgery (e.g. seeing with an interocular lense) or residual visual impairments, we included six individuals with a history of either developmental or incomplete congenital cataracts (refered to as developmental cataract individuals, dc). Moreover an additional group of healthy age matched controls (mc) was tested. It was recently shown that congenital cataract individuals and matched controls display a similar modulation of the N1 of the ERPs while processing biological motion^20^. Since phase-locked theta oscillatory activity has been related to the N1, we predicted evoked theta oscillatory activity to vary as a function of biological motion processing in all groups. By contrast, alpha activity was hypothesized to depend on visual input from birth.

## Results

### Behavioral recovery

**Figure 1:**
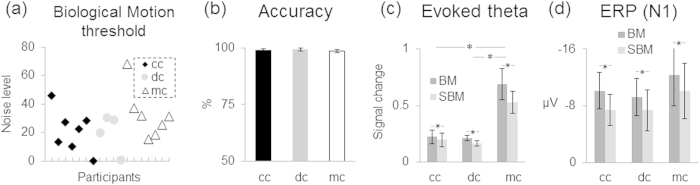
Behavioral and EEG/event-related brain potential (ERP) results. (**a**) Thresholds of single participants (black diamond = congenital cataract group, cc; gray dots = developmental cataract group, dc; white triangles = matched controls, mc) in the behavioral biological motion (BM) detection task^22^; higher values indicate higher sensitivity. The three groups did not differ in detecting BM. (**b**) Hit rates in the EEG biological motion task are separately shown for all groups. The three groups did not differ in their ability to detect the visual targets. (**c**) Relative power change of the evoked theta response (4–7 Hz; averaged across posterior electrodes, TP8/9, P7/8, and O1/2). Data are shown separately for each group (cc, dc, mc) and condition (BM and scrambled biological motion, SBM). Evoked theta activity was higher for BM than for SBM in all three groups; in addition, the mc group showed higher evoked theta response compared to both dc and cc groups. (**d**) Mean amplitudes of the N1 wave of the event-related potentials elicited by BM and SBM stimuli, displayed separately for the cc, dc and mc groups. The N1 was enhanced to biological compared to scrambled biological motion stimuli irrespectively of group (data of cc and mc groups from^20^). *indicates significant condition and/or group effects.

We ran the subtest “detection” of the Motion Lab Battery^22^, which is a standardized assessment of the ability to detect biological motion stimuli (point light displays of walking humans) in subgroups of our participants since data was not available for all due to time limitations and technical problems (see Material and Methods). This test did not reveal any group difference (group effect (F(2, 15) = 1.2, p > 0.3) (Fig. 1a): The cc group’s (n = 7, see Table 1) performance did neither differ from the performance of the mc group (n = 7; t(12) = −1.3, p > 0.2) nor from the performance of the dc group (n = 4, see Table 1; t(9) = 0.1, p > 0.9), and the dc group did not differ from the mc group (t(9) = 1.2, p > 0.2).

### Biological Motion experiment

#### Behavioral performance

While recording the EEG, intact and scrambled point light displays of walking humans (biological motion, BM, and scrambled biological motion, SBM, respectively) were presented intermixed with rare “target” point light displays of a moving cat which had to be detected. All groups (cc: N = 12, dc: N = 6 and mc: N = 12, see Table 1) performed at a high level and no difference in detection rates between groups was observed (mean hit rates: cc = 98.8%, SE = 0.6, dc = 99.3%, SE = 0.7 and mc = 98.6%, SE = 1.0 and; F(2, 26) = 0.1, p > 0.8, Fig. 1b). The false alarms rate was below 1% in all groups.

#### EEG results

Oscillatory brain activity in the theta (4–7 Hz) and in the alpha band (8–12 Hz) were analyzed. The results for the evoked theta activity replicated previous ERP-N1 findings (see^23^): Higher theta power was observed for intact than for scrambled biological motion stimuli (main effect of condition F(1, 27) = 10.9, p = 0.003). This effect was observed in each group (cc: t(11) = 2.4, p = 0.03; dc: t(5) = 2.5, p = 0.055; mc: t(11) = 3.0, p = 0.01; see Fig. 1c and supplementary Figure 1). Theta band power was, however, overall higher in the mc group than in the cc group and the dc group (main effect of group F(2, 27) = 6.9, p < 0.01; mc vs. cc: p < 0.01, mc vs. dc: p < 0.03), while the cc group and dc group did not differ (p > 0.8).

**Figure 2:**
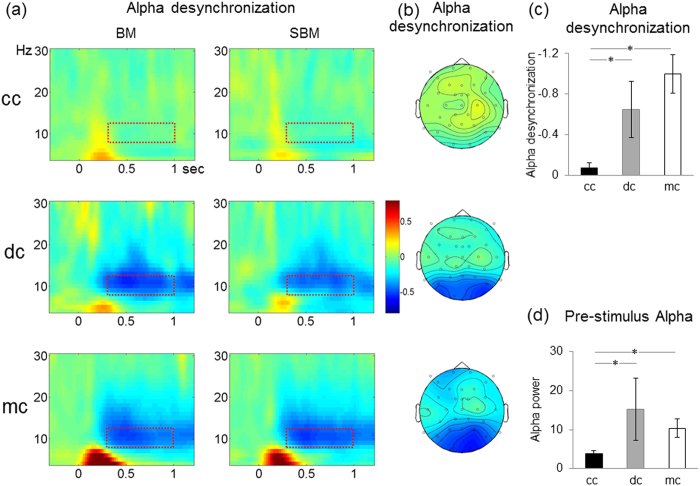
Alpha activity. (**a**) Time frequency representation of the relative power signal change (4–30 Hz, total power, see methods; averaged across posterior electrodes and hemispheres, TP8/9, P7/8, and O1/2) with respect to baseline. Data are shown separately for each group (cc, dc, mc) and condition (biological motion, BM and scrambled biological motion, SBM). (**b**) Scalp topographies of the alpha desynchronization (decrease of the power between 8–12 Hz with respect to baseline in time epoch 0.3–1 sec) after the stimulus onset is plotted separately for each group. (**c**) Bar plots of the mean alpha desynchronization (8–12 Hz; time window: 0.3–1 sec, see red dotted box in (**a**)), with standard errors is displayed for each group. The dc and mc groups showed a significantly higher alpha desynchronization compared to the cc group. (**d**) Pre-stimulus alpha level (non-baseline corrected) with standard errors plotted separately for each group. The alpha activity of the cc group was significantly reduced. *indicates significant condition and/or group effects.

Alpha desynchronization (that is, post-stimulus onset alpha oscillatory activity compared to baseline) significantly differed between groups (main effect of group; F(2, 27) = 10.6, p < 0.001; Fig. 2a–c). The alpha desynchronization was stronger in both the mc and the dc group compared to the cc group, (t(22) = −4.9, p < 0.001 and t(16) = −2.7, p < 0.02, respectively). By contrast, the mc and the dc group did not differ (t(16) = −1.4, p > 0.1). As seen in the scalp topographies (Fig. 2b), the dc and the mc groups displayed an extended posterior alpha desynchronization while a similar modulation was not observed in the cc group at any of the recording sites.

Finally, comparing post-stimulus alpha power to zero in each group revealed a significant desynchronization in the mc group (t(11) = −5.4, p < 0.001, one tailed) as well as in the dc and the cc groups (t(5) = −2.3, p < 0.05, one tailed; t(11) = −1.8, p < 0.05, one tailed respectively).

**Figure 3:**
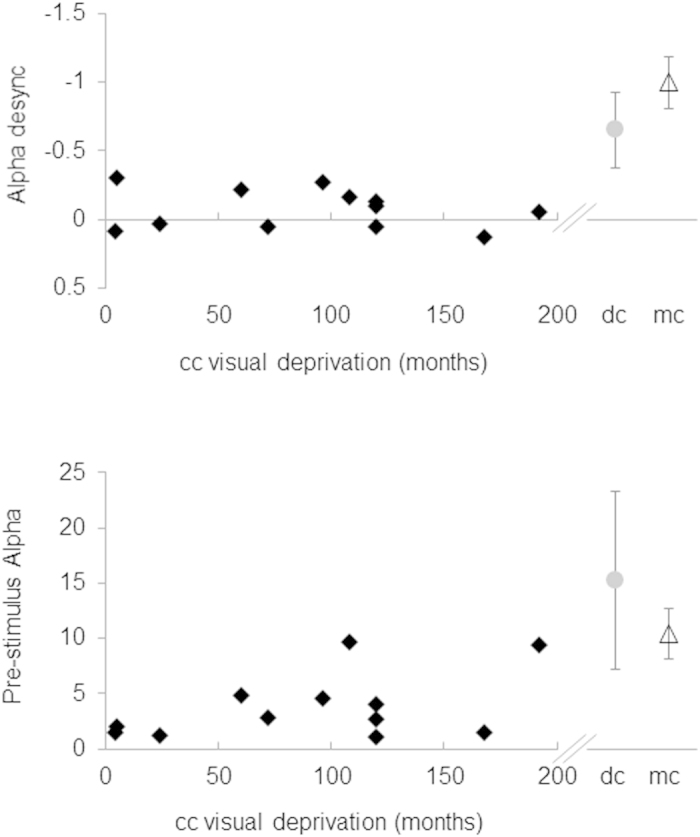
Degree of alpha desynchronization (upper panel, see Fig. 2) and degree of alpha pre-stimulus (lower panel, see Fig. 2) in single participants. The data of the cc are shown as a function of the age at surgery (left part of the panels). For comparison the data of the dc and mc participants are displayed on the right.

The degree of alpha desynchronization in the cc group was independent of the duration of visual deprivation (r = 0.2, p > 0.5, see Fig. 3), the time since surgery (r = 0.2, p > 0.4) and the achieved visual acuity (r = −0.06, p > 0.8).

In none of the three groups an effect of condition (BM vs. scrambled BM) was observed for the degree of alpha desynchronization (main effect of condition F(1, 26) = 0.2, p > 0.6). To test whether overall alpha activity was reduced in the cc group or whether only the degree of alpha desynchronization (baseline vs. post-stimulus alpha level, the “alpha blocking”) was altered, the average alpha activity recorded in the pre-stimulus period [-200-0ms] was compared between groups. The marginally significant group effect (F(2, 27) = 2.6, p = 0.09) was due to a reduced pre-stimulus alpha activity in the cc compared to the mc group (t(22) = 2.7, p < 0.01; one-tailed) and the dc group (t(16) = 1.9, p < 0.04; one-tailed; Fig. 2d). By contrast the dc and mc group did not differ (t(16) = −0.6, p > 0.5).

## Discussion

The present study evaluated neural mechanisms of sensitive period plasticity in humans. We assessed oscillatory activity in the human electroencephalography within the theta and alpha band during the processing of intact and scrambled biological motion stimuli. The alpha desynchronization as well as the pre-stimulus alpha activity was significantly reduced in congenital cataract-reversal individuals (cc) compared to both control groups, that is, individuals with a history of developmental or incomplete congenital cataracts (dc) and normally sighted controls (mc). By contrast, evoked theta oscillatory activity varied with biological motion processing in all three groups. Overall, theta activity was lower in the cc and dc group compared to the mc group. At the behavioral level the three groups were indistinguishable in their sensitivity to detect biological motion stimuli.

The lack of group differences in biological motion processing has now been demonstrated in two independent studies, in which intact performance has been found after a short period of visual deprivation of a few weeks to months^19^ and s after long periods of deprivation lasting for several years^20^. To increase the power, Hadad *et al.*^19^ employed a control group comprising 80 healthy individuals. Nevertheless, zero results are always tentative and need additional replication in independent and if possible larger samples. Moreover, other types of biological motion stimuli need to be tested.

In the cc group as well as the dc and mc groups evoked theta band power varied with biological motion processing. This result confirms recent findings of our group (in normally sighted controls and in cc individuals^20^) demonstrating, as previously reported in healthy individuals^24^,^25^,^26^,^27^ larger N1 amplitudes for intact compared to scrambled biological motion stimuli. It is known that the evoked theta oscillatory activity and the N1 amplitude are highly correlated^23^ and that evoked theta oscillations partially show up as the P1-N1 complex observed in the ERPs^23^. Since we did not observe a difference between groups in detecting biological motion stimuli, the present results suggest that the recovery of biological motion processing^19^,^20^, at least the features assessed in the present tasks, might or might partially be associated with a recovery of visually evoked theta activity and related neural mechanisms. We further conclude that these mechanims are not related to a sensitive phase in visual development. The only group difference we found for the theta activity was an overall lower power in both the cc and the dc group compared to the mc group. Although we had not observed similar group differences in the ERPs^20^, we speculate that these differences are possibly due to prevailing visual impairments in the cataract groups (both the cc and dc group). Importantly, the effects of the experimental manipulation were indistinguishable across all groups suggesting, in accord with the behavioral results, that all participants were able to distinguish the scambled and intact biological motion stimuli.

Both, stimulus induced alpha desynchronization as well as overall prestimulus alpha activity were significantly impaired in the cc group compared to both the dc and the mc group. First, this result pattern allows us to exclude any unspecific factors such as having a history of cataracts, cataract surgery, the use of interocular lenses and remaining visual impairments as possible accounts for the observed group differences. Moreover, since the cc and dc individuals were recruited from the same population and examined in the same laboratory space, we are able to discount any unspecific effects of ethnicitiy, social brackground and recording environment.

Animal studies have shown that even minimal visual experience is sufficient to induce a typical functional and structural organization of the brain, whereas the lack of such inputs immediately after birth seems to result in permanent impairments^28^. This finding is in accord with our results: We demonstrate that the, though limited, patterned visual input available to individuals with a history of congenital but incomplete cataracts was sufficient to set up the neural mechanisms for alpha generation and thus the neural mechanisms important for the control of visual cortex activity^1^,^2^. Indeed, Novikova^11^ reported a lack of alpha activity only in individuals with a congenital visual impairment allowing for no more than light perception. Thus, we assume that the individuals with incomplete congenital and the individuals with developmental cataracts all had set up the neural circuits important for alpha generation whereas the cc individuals, had been lacking any structural visual input from birth, had not. In this context, it is important to stress that the assignment of congenital cataract individuals to the cc and the dc group was based on the medical records and was completed prior to the start of the data analysis.

Since alpha oscillations depend on inhibitory circuits that allow for the control of the excitatory/inhibitory balance of neural networks, the present data might additionally support the conclusion that this hallmark of functional brain development^21^ is experience dependent in humans as well. Implementing and stabilizing inhibition in neural circuits are known as crucial mechanisms of sensitive phase plasticity^18^ and go together with the functional specialization of neural networks^29^. Initial evidence has demonstrated that after a period of visual deprivation from birth the neural systems engaged in face processing in healthy individuals were similarly activated by other visual stimulus categories (e.g. houses) in cc individuals^29^,^30^. Possibly, as a result of this lack of functional specialization, deficits in face processing, known for several years^31^, emerged. It may even be speculated that other deficits observed in cc individuals, including deficits in visual acuity^16^,^32^ and contrast sensitivity^33^, visual feature binding^34^,^35^ as well as in the recognition of static objects^36^,^37^, might result from a similar lack of functional specialization of the associated brain regions.

Since alpha activity was reduced in the prestimulus baseline as well, we speculate that reduced posterior alpha activity in cc individuals as known for permanently blind individuals^9^,^10^,^11^,^12^ indicates a general processing deficit in the congenitally visual deprived visual cortex even after restoring sight.

As previously shown^38^,^39^ alpha activity did not vary with biological motion processing in the normally sighted control group of the present study. Given this observation along with the typical modulation of theta activity in the cc group, it is, thus, not surprising that we did not observe behavioral deficits in this group. However, we predict that every visual task that depends on the activity regulation of alpha oscillatory activity should be impaired to some degree in the cc but not to the same degree in the dc group. Indeed, cc individuals display impairments in global motion processing^19^, which has been related to alpha activity^40^.

Furthermore, alpha activity has often been demonstrated to play a role in the control of overt and covered attention^41^. Interestingly, the pyramidal cells in layer V of the primary visual cortex maintain strong connections to the superior colliculi (SC), which plays an essential role in the control of eye movements^42^. In animals, the direct visual input to the SC was found to be largely reduced after total visual deprivation from birth. Moreover, the cortico-tectal connectivity seemed to have completely failed^43^. Indeed, visual responses in the SC were markedly reduced after congenital visual deprivation^44^. Therefore, we expect the cc group to show impaired in both overt and covert visual attention control. Indeed, there is first evidence from a visual-spatial attention task supporting this hypothesis^45^.

Finally, it must be wondered why posterior alpha activity was more strongly impaired in cc individuals than visually evoked theta activity, considering that they both depend on GABA-mediated inhibition. There is evidence, however, that different and partially independent inhibitory circuits exist for the generation of low (theta) and higher frequency oscillations^46^. Our data, thus, suggest that these different inhibitory circuits show different degrees of experience-dependence and that particularly the setting up of fast spiking PV (Parvalbumin) interneurons, which are important for alpha generation^1^ and possibly gain control^47^, are experience-dependent during a sensitive phase of development (see^48^). In this context, it is important to remember that the newborn EEG includes theta oscillations^7^ while posterior alpha activity does not seem to emerge before late childhood^8^.

In sum, our results demonstrate that the recovery of biological motion processing goes together with a reinstatement of visually evoked theta activity suggesting that the acquisition of the involved neural mechanisms is not linked to a sensitive phase in development. By contrast, the emergence of posterior alpha oscillations and their underlying neural circuits seem to require visual input during a sensitive phase in development. Thus based on the present findings new experiments can be designed to test the specific functional consequences of altered oscillatory brain activity following a transient phase of congenital blindness.

## Material and Methods

### Participants

The group of individuals with a transient phase of blindness from birth comprised a total of 12 individuals (4 females) with a history of congenital, bilateral, dense cataracts (congenital cataract individuals, cc; mean age: 17.8 years, range: 11–35, see Table 1), recruited at the LV Prasad Eye Institute in Hyderabad (India). The history of cataracts was confirmed by medical records. Since the cataracts were diagnosed at different ages, additional criteria were applied to guarantee that only individuals with congenital total cataracts entered this group. These criteria included: the presence of nystagmus, density of the lenticular opacity, invisibility of the fundus prior to surgery, family history and family reports. Most of the participants had only light perception prior to surgery (see Table 1). On average cc individuals underwent surgery at the mean age of 94 months (range: 4–192). The duration since surgery was on average 119 months (range: 12–396). The visual acuity at the time of testing (as assessed during the same visit in 8 participants, within 4 months prior of testing in 3 participants and within 18 months in 1 participant) was 0.14 decimals (range: 0.05–0.50). Due to incomplete datasets, a subset of 7 individuals (3 females, mean age: 18.5 years, range: 10–35, mean visual acuity: 0.2 decimals, range: 0.05–0.50; mean age at surgery: 86 months, range: 4–168, see Table 1) were tested in a behavioral biological motion detection task.

An additional group of 6 individuals who had suffered from developmental cataracts (cataracts were not present at birth and developed later) or congenital but incomplete cataracts (cataracts were not fully dense at birth and allowed patterned light to reach the retina) was recruited (dc) at the same institute (4 females, mean age: 17.4 years, range: 8–38, mean visual acuity: 0.2 decimals, range: 0.08–0.80; mean age at surgery: 106 months, range: 24–252; see Table 1). The mean age at surgery was 88 months (range: 24–252); duration since surgery was 68.5 months (range: 12–204). The mean achieved acuity (as assessed at the time of testing in 3 participants, within 4 months prior of testing in 2 participants and within 18 months in 1 participant) was 0.3 decimals (range: 0.08–0.80). Due to incomplete datasets, a subset 4 individuals were tested behaviorally in the biological motion detection task (2 females, mean age: 10.8 years, range: 8–17, mean visual acuity: 0.3 decimals, range: 0.08–0.80, mean age at surgery: 69 months, range: 24–96; see Table 1). Post-surgical visual acuity at the latest assessment did not differ between the cc and the dc groups in any of the subsamples participating in the two tasks (biological motion EEG: t(17) = 1.6, p > 0.14; biological motion detection behavioral experiment: t(9) = 0.9, p > 0.41). Note, that individuals were assigned to the cc or dc groups prior the data analysis.

Incomplete data sets resulted from technical errors or time constraints of the participants.

All cc and dc participants were right handed and neurologically healthy according to self-report and medical examination by a physician.

For each of the two experiments, a sample of healthy control (matched controls, mc) participants matched in age sex and handedness was recruited in Hamburg, Germany. All mc participants had normal or corrected to normal vision and were neurologically healthy according to self-report. A sample of 12 control individuals participated in the biological motion EEG experiment (6 females, mean age: 18 years, range: 8–37); an additional sample of 7 individuals (in order to match the cc group) participated in the behavioral biological motion detection task (3 females mean age: 19.0 years, range: 10–27).

Participants and, for minors, their legal guardians gave written informed consent after the nature of the study was explained. The methods were carried out in accordance with the approved guidelines and the study was approved by the ethical committee of the German Society of Psychology and the ethical committee of the LV Prasad Eye institute.

### EEG experiment

Three categories of dynamic motion stimuli were randomly presented: (1) intact walkers, (2) scrambled walkers, and (3) a walking cat (for a detailed description of the stimuli see^49^. The point-light displays were presented in profile view, facing either to the left or to the right. The walker was created by the movement of 11 white squares presented on a black background. Each point-light had a width of 0.3°. The height of the walker subtended 8.6°, and the width was 4.3°. In the scrambled motion version, each single point-light trajectory was randomly swapped with the location of another trajectory, so that, for instance, a marker that featured the movement of an elbow appeared at the location of the knee marker etc. This scrambling procedure preserves the overall shape of the walker while scrambling local motion and insures that the size of the two stimulus categories matches. The starting phase of each walker was randomized as well, so that incidental shape differences between intact and scrambled walkers were averaging out. Eight different walkers were created, four facing left, four right. During the locomotion the walker remained at the same central location. Point-light displays depicting the profile view of a walking cat (four facing left, four right) were used as behavioral targets. The target’s height subtended 4.2° and the width was 7.9°. Each stimulus category was presented for 2000 ms. The inter-trial interval (ITI) ranged pseudo-randomly from 2000 to 2700 in steps of 100 ms. A total of 48 target trials (p = 0.17) were randomly intermixed with 240 trials (p = 0.83), half with intact and half with scrambled walkers. A total of 6 blocks were presented (48 trials each). Participants were asked to detect the walking cat, responding via mouse button press or verbally.

### Behavioral biological motion detection task

Biological motion detection thresholds were assessed with an adapted version of the subtest “Detection test” of the Biological Motion Perception Test Battery (BML test battery^22^). Larger dot and display sizes were implemented (15 pixels and 10.5 × 10.5°). One stimulus consisted of a walker made out of point-lights^50^ superimposed by a mask of scrambled walker noise. The walker was shown with an offset from the center point of the mask that varied randomly between −0.5 and 0.5 degrees of visual angle horizontally and between −1.5 and 1.5 vertically. The second display comprised the same number of dots but only scrambled walker motion was presented. Participants’ task was to indicate whether a walker was presented in the first or in the second display. After the response, blank screen was displayed for 1 sec before the next trial started. The number of masking noise dots was set by a QUEST procedure^51^. The detection threshold was defined as the number of noise dots at which the participant achieved a hit rate of 82%. The test terminated after 40 trials^52^.

### Stimuli and apparatus

Stimuli were presented with a Dell laptop on a Dell 22 inches LCD monitor with a refresh rate of 60 Hz and were created with MatLab^©^ and the psychotoolbox 3 software^53^,^54^. Stimulus presentation in the EEG experiment was controlled using the Presentation^®^ software (http://www.neurobs.com/) Participants sat at a distance of 60 cm from the screen and were instructed to keep their head and eyes oriented towards fixation throughout testing (one experimenter was always sitting beside the participant to ensure compliance with the task instructions. If necessary a translator instructed the participants in their native language. Prior to the experimental trials, one additional block was run as practice. The total experiment, including the EEG task, EEG application and removal, and the behavioral task, took on average 2 h.

### EEG recording

The EEG was continuously recorded (analog bandwidth: 0.01–200 Hz, sampling rate: 500 kHz, BrainAmp, http://www.brainproducts.com/) with 30 Ag/AgCl electrodes attached to an elastic cap (Easy cap) at standard 10–20 sites including Fp1-Fp2-F7-F3-Fz-F4-F8-T7-C3-Cz-C4-T8-P7-P3-Pz-P4-P8-O1, and O2. Additional electrodes at intermediate sites were mounted at FC5-FC1-FC2-FC6-TP9-CP5-CP1-CP2-TP10-F9 and F10. All scalp recordings were performed against the right ear lobe. Horizontal eye movements were monitored with a bipolar montage comprising two electrodes close to the left and right outer canthi of the eyes (F9-F10) and Vertical eye-movements with frontal electrodes (Fp1-Fp2). Offline, data were down sampled to 250 Hz and re-referenced to an average reference for the time-frequency analysis (theta and alpha) and to Fz for the ERP analysis^55^,^56^. To eliminate artifacts related to eye movements and heart beat we performed an Independent Component Analysis^57^ runica version, implemented on EEGLAB^57^ (running in MatLab^®^). In addition, trials with signals exceeding 100 μV (130 μV for participant cc-e and f) were eliminated prior to averaging. For all participants, at least 40% artifact free trials for both ERP and EEG analyses remained after the artifact elimination. EEG analyses were conducted for the standard stimuli which did not require any response. Electrophysiological recordings were analyzed with the EEGLAB^58^ and fieldtrip software^59^.

### Spectral analysis

The data analyses concentrated on the alpha oscillatory activity (8–12 Hz; total power) since we had clear a priori predictions. The evoked theta activity (4–7 Hz) was assessed as well, since it allowed us to link our findings to the event-related potential literature on biological motion processing^23^. A reliable assessment of higher frequency bands (such as gamma activity) was not possible because we had to record in an unshielded room at the LV Prasad Eye Institute and because it was not possible to record a sufficient number of trials per condition due to the limited available time for recording.

The continuous EEG was segmented into 3000 ms epochs, including 1000 ms preceding the stimulus onset.

### Total power

Spectral changes in oscillatory activity were analyzed using a wavelet transform, which provides a good compromise between time and frequency resolution^60^. Time–frequency analyses were performed for each channel by convolving the data with a complex Morlet wavelet (t,f0) which had a Gaussian shape in time (σt) and in frequency (σf) around the center frequency (f0). Non-constant wavelets with Q increasing from f0/σf = 4 to 8.5 for frequencies from 2 to 40 Hz (step size 1 Hz) were employed. The wavelet transform was performed between −800 to 2000 ms post stimulus in steps of 20 ms. Importantly, the transformation of the data was done before averaging the single trials, separately for each frequency. For this reason the total power represents both the phase locked (evoked) and the non-phase locked signal with respect to the event. The resulting power was baseline corrected for each frequency to obtain the relative signal change: P(t,f)_corrected_ = (P(t,f)_post-stimulus_ − P(f)_baseline_)/P(f)_baseline_. The pre-stimulus period between −700 to −300 ms served as baseline for all spectral analyses. This baseline prevented including slow frequency activity emerging immediately before the stimulus onset. Grand mean time-frequency representations were computed across all participants to illustrate the relative change of activity with respect to the baseline across frequencies. Total power activity was calculated for each electrode to assess alpha desynchronization.

### Evoked power

The same time-frequency transformation was applied after averaging across trials. The average signal represents only the phase locked signal. To compute the relative signal change of evoked power, data were normalized with respect to the total power baseline as follows: P(t,f)_evoked_ = (P(t,f)_evoked_ − P(f)_evoked-baseline_)/P(f)_total-baseline_. The evoked power time frequency decomposition provided the data for the analysis on the evoked theta (see supplementary Figure 1).

### Statistical approach

#### Spectral analysis of the alpha activity

Post-stimulus onset (alpha desynchronization): baseline corrected alpha total power was extracted for each individual and averaged within a 700 ms time window [300–1000ms]. Since the alpha desynchronization is observed over a large area of the posterior scalp, the relative change in alpha power with respect to baseline was averaged separately for each hemisphere across the posterior electrodes TP8/9, P7/8, and O1/2. Relative alpha power was then used as dependent variable for a repeated measurement ANOVA with the between participant factor group (cc, mc and dc) and the repeated measurement factors hemisphere (left and right) and condition (BM and SBM). Main effects of group were followed up by two tailed (one-tailed t-tests are explicitly marked) t-tests (mc vs. cc; dc vs. cc; mc vs. dc).

Additionally, it was tested whether a significant desynchronization existed in each group by comparing the post-stimulus alpha power against zero.

Pre-stimulus alpha activity, BM EEG: For each participant, the alpha power (without baseline correction) was extracted and averaged across a 200 ms time window [-200-0 ms]. The alpha power was averaged across posterior electrodes (see post-stimulus alpha desynchronization). This score was entered as dependent variable to a repeated measurement ANOVA analogue to the analysis performed on post-stimulus onset data.

#### BM evoked theta activity

To evaluate the evoked theta activity, we adopted a similar analysis strategy as for the ERP. The most prominent positive peak was identified across all electrodes for each individual within the time window of 160 to 260 ms. The theta response was then averaged within a 64 ms time window centered at the individual evoked theta peak. The average was computed for each electrode and condition for each participant. Mean theta power served as dependent variable in a repeated measurement ANOVA with the between participant factor group (cc, mc, and dc) and the repeated measurement factors electrode location (temporo-occipital (TP9/10), parieto-occipital (P7/8), and occipital (O1/2)), hemisphere (left and right) and condition (BM and SBM).

## Additional Information

**How to cite this article**: Bottari, D. *et al.* Sight restoration after congenital blindness does not reinstate alpha oscillatory activity in humans. *Sci. Rep.* **6**, 24683; doi: 10.1038/srep24683 (2016).

## Supplementary Material

Supplementary Information

## Figures and Tables

**Table 1:** Description of participants and performance in the four different tasks.

Participant	Age (years)	Gender	Cataract onset	Age at surgery (months)	Fundus visibility pre-surgery best eye	Nystagmus	Presurgical visual acuity in best eye*	Last postsurgical visual acuity in best eye		Mean accuracy in BM EEG task, %	BM detection task, noise dots n**
								Decimal	logMar		
cc-a	23	M	congenital	48	Unknown	yes	Unknown	0.16	0.80	100.0	46.3
cc-b	35	M	congenital	24	Unknown	yes	Unknown	0.50	0.30	93.8	13.7
cc-c	17	F	congenital	168	No view	yes	FC:0.5	0.13	0.90	100.0	–
cc-d	17	F	congenital	192	No view	yes	PL + , PR +	0.02	1.78	97.8	–
cc-e	10	M	congenital	108	No view	yes	PL + , PR +	0.02	1.78	100.0	–
cc-f	11	F	congenital	120	No details	yes	FC:0.5	0.05	1.30	96.4	0.2
cc-g	31	M	congenital	72	Unknown	yes	No vision	0.05	1.30	100.0	27.4
cc-h	11	F	congenital	120	No view	yes	PL + , PR +	0.16	0.80	100.0	10.2
cc-i	11	M	congenital	96	No view	yes	PL + , PR +	0.32	0.50	100.0	22.7
cc-l	13	M	congenital	120	No view	yes	PL+, PR+	0.02	1.78	100.0	28.7
cc-m	21	M	congenital	4	Unknown	yes	Unknown	0.13	0.90	100.0	–
cc-n	13	M	congenital	60	No view	yes	Unknown	0.13	0.90	97.4	–
**Mean**	**17.8**			**94.3**				**0.14**	**1.09**	**99.8**	**21.3**
dc-a	19	F	Developmental	24	Unknown	yes	PL+, PR+	0.80	0.10	100.0	20.1
dc-b	8	M	developmental	84	Unknown	no	0.25	0.25	0.60	100.0	0.9
dc-c	22	F	congenital not dense	252	Haze but Visible	yes	FC:0.5	0.08	1.10	100.0	–
dc-e	38	F	congenital not dense	Not operated	Visible	yes	0.08	0.30	1.10	100.0	–
dc-g	10	F	congenital not dense	72	No view	no	FC:2	0.13	0.90	95.8	30.6
dc-h	9	M	congenital not dense	96	Haze but Visible	yes	FC:1	0.08	1.10	100.0	28.9
**Mean**	**17.6**			**88.0**				**0.27**	**0.82**	**99.3**	**20.1**

^*^PL+: able to perceive light; PR+: able to report the location of light; FC: able to count fingers at n meters.

BM: Biological motion; GM: Global motion.

^**^Degree if noise at which a hit rate of 82% was reached (high values indicate high sensitivity) in the behavioral BM task.

^***^Percentage of coherence level necessary to reach a hit rate of 82% (high values indicate low sensitivity) in the behavioral GM task.

Note patient dc-a was categorized as dc due to parents’ reports supported by a low nystagmus and the achieved high visual outcome.

## References

[b1] Jensen O., Bonnefond M. & VanRullen R. An oscillatory mechanism for prioritizing salient unattended stimuli. Trends in Cognitive Sciences 16, 200–206, doi: 10.1016/j.tics.2012.03.002 (2012). 22436764

[b2] Klimesch W. Alpha-band oscillations, attention, and controlled access to stored information. Trends in Cognitive Sciences 16, 606–617, doi: 10.1016/j.tics.2012.10.007 (2012). 23141428 PMC3507158

[b3] Ergenoglu T. *et al.* Alpha rhythm of the EEG modulates visual detection performance in humans. Cognitive Brain Research 20, 376–383, doi: 10.1016/j.cogbrainres.2004.03.009 (2004). 15268915

[b4] van Dijk H., Schoffelen J. M., Oostenveld R. & Jensen O. Prestimulus oscillatory activity in the alpha band predicts visual discrimination ability. Journal of Neuroscience 28, 1816–1823, doi: 10.1523/jneurosci.1853-07.2008 (2008). 18287498 PMC6671447

[b5] Kelly S. P., Lalor E. C., Reilly R. B. & Foxe J. J. Increases in alpha oscillatory power reflect an active retinotopic mechanism for distracter suppression during sustained visuospatial attention. Journal of Neurophysiology 95, 3844–3851, doi: 10.1152/jn.01234.2005 (2006). 16571739

[b6] Buffalo E. A., Fries P., Landman R., Buschman T. J. & Desimone R. Laminar differences in gamma and alpha coherence in ventral stream. PNAS 108, 11262–11267 (2011). 21690410 10.1073/pnas.1011284108 PMC3131344

[b7] Eisermann M., Kaminska A., Moutard M. L., Soufflet C. & Plouin P. Normal EEG in childhood: From neonates to adolescents. Neurophysiologie Clinique/Clinical Neurophysiology 43, 35–65, doi: 10.1016/j.neucli.2012.09.091 (2013). 23290174

[b8] Başar E. A review of alpha activity in integrative brain function: fundamental physiology, sensory coding, cognition and pathology. International Journal of Psychophysiology 86, 1–24, doi: 10.1016/j.ijpsycho.2012.07.002 (2012). 22820267

[b9] Berger R. J., Olley P. & Oswald I. The EEG, eye-movements and dreams of the blind. Quarterly Journal of Experimental Psychology 14, 183–186, doi: 10.1080/17470216208416534 (1962).

[b10] Novikova L. A. Blindness and the electrical activity of the brain. American foundation for the Blind research Series 23 (1973).

[b11] Schubert J. T., Buchholz V. N., Föcker J., Engel A. K., Röder B. & Heed T. Oscillatory activity reflects differential use of spatial reference frames by sighted and blind individuals in tactile attention. Neuroimage. 15(117) 417–28, doi: 10.1016 (2015). 10.1016/j.neuroimage.2015.05.068 26032885

[b12] Kriegseis A., Hennighausen E., Rösler F. & Röder B. Reduced EEG alpha activity over parieto-occipital brain areas in congenitally blind adults. Clinical Neurophysiology 117, 1560–1573, doi: 10.1016/j.clinph.2006.03.030 (2006). 16759908

[b13] Hawellek D. J. *et al.* Altered Intrinsic neuronal interactions in the visual cortex of the blind. Journal of Neuroscience 33, 17072–17080, doi: 10.1523/jneurosci.1625-13.2013 (2013). 24155311 PMC6618438

[b14] Maurer D., Lewis T. & Mondloch C. Missing sights: consequences for visual cognitive development. Trends in Cognitive Sciences 9, 144–151, doi: 10.1016/j.tics.2005.01.006 (2005). 15737823

[b15] Lewkowicz D. J. & Röder B. Development of multisensory processing and the role of early experience. (MIT Press., 2012).

[b16] Ganesh S. *et al.* Results of late surgical intervention in children with early-onset bilateral cataracts. British Journal of Ophthalmology 98, 1424–1428, doi: 10.1136/bjophthalmol-2013-304475 (2014). 24879807 PMC4841630

[b17] Wiesel T. N. & Hubel D. H. Comparison of the effects of unilateral and bilateral eye closure on cortical unit response in kittens. Journal of Neurophysiology 28, 1029–1041 (1965). 5883730 10.1152/jn.1965.28.6.1029

[b18] Levelt C. N. & Hübener M. Critical-period plasticity in the visual cortex. Annual Review of Neuroscience 35, 309–330, doi: 10.1146/annurev-neuro-061010-113813 (2012). 22462544

[b19] Hadad B.-S., Maurer D. & Lewis T. L. Sparing of sensitivity to biological motion but not of global motion after early visual deprivation. Developmental Science 15, 474–481, doi: 10.1111/j.1467-7687.2012.01145.x (2012). 22709397

[b20] Bottari D. *et al.* The neural development of the biological motion processing system does not rely on early visual input. Cortex 71, 359–367, doi: 10.1016/j.cortex.2015.07.029 (2015). 26301874

[b21] Hensch T. K. Critical Period Regulation. Annual Review of Neuroscience 27, 549–579, doi: 10.1146/annurev.neuro.27.070203.144327 (2004). 15217343

[b22] Saunders D. R. & Troje N. F. A test battery for assessing biological motion perception. Journal of Vision 11, 686–687 (2011).

[b23] Klimesch W. *et al.* Phase-locked alpha and theta oscillations generate the P1–N1 complex and are related to memory performance. Cognitive Brain Research 19, 302–316, doi: 10.1016/j.cogbrainres.2003.11.016 (2004). 15062867

[b24] Hirai M., Senju A., Hirokata F. & Hiraki K. Active processing of biological motion perception: an ERP study. Cognitive Brain Research 23, 387–396, doi: 10.1016/j.cogbrainres.2004.11.005 (2005). 15820645

[b25] Krakowski A. I. *et al.* The neurophysiology of human biological motion processing: a high-density electrical mapping study. NeuroImage 56, 373–383, doi: 10.1016/j.neuroimage.2011.01.058 (2011). 21276862 PMC6589837

[b26] Jokisch D., Daum I., Suchan B. & Troje N. Structural encoding and recognition of biological motion: evidence from event-related potentials and source analysis. Behavioural Brain Research 157, 195–204, doi: 10.1016/j.bbr.2004.06.025 (2005). 15639170

[b27] Hirai M., Watanabe S., Honda Y. & Kakigi R. Developmental changes in point-light walker processing during childhood and adolescence: an event-related potential study. Neuroscience 161, 311–325, doi: 10.1016/j.neuroscience.2009.03.026 (2009). 19303916

[b28] Innocenti G. M., Frost D. O. & Illes J. Maturation of visual callosal connections in visually deprived kittens: challenging critical period. The Journal of Neuroscience 5, 255–267 (1985). 3973665 10.1523/JNEUROSCI.05-02-00255.1985 PMC6565202

[b29] Röder B., Ley P., Shenoy B. H., Kekunnaya R. & Bottari D. Sensitive periods for the functional specialization of the neural system for human face processing. Proceedings of the National Academy of Sciences 110, 16760–16765, doi: 10.1073/pnas.1309963110 (2013). PMC3801039 24019474

[b30] Grady C. L., Mondloch C. J., Lewis T. L. & Maurer D. Early visual deprivation from congenital cataracts disrupts activity and functional connectivity in the face network. Neuropsychologia 57, 122–139, doi: 10.1016/j.neuropsychologia.2014.03.005 (2014). 24657305

[b31] Grand R. L., Mondloch C. J., Maurer D. & Brent H. P. Expert face processing requires visual input to the right hemisphere during infancy. Nature Neuroscience 6, 1108–1112, doi: 10.1038/nn1121 (2003). 12958600

[b32] Maurer D. & Lewis T. L. Visual acuity: the role of visual input in inducing postnatal change. Clinical Neuroscience Research 1, 239–247, doi: 10.1016/S1566-2772(01)00010-X (2001).

[b33] Kalia A. *et al.* Development of pattern vision following early and extended blindness. Proceedings of the National Academy of Sciences 111, 2035–2039, doi: 10.1073/pnas.1311041111 (2014). PMC3918801 24449865

[b34] Putzar L., Hötting K., Rösler F. & Röder B. The development of visual feature binding processes after visual deprivation in early infancy. Vision Research 47, 2616–2626, doi: 10.1016/j.visres.2007.07.002 (2007). 17697691

[b35] McKyton A., Ben-Zion I., Doron R. & Zohary E. The Limits of Shape Recognition following Late Emergence from Blindness. Current Biology 25, 2373–2378, doi: 10.1016/j.cub.2015.06.040 (2015). 26299519

[b36] Fine I. *et al.* Long-term deprivation affects visual perception and cortex. Nature Neuroscience 6, 915–916, doi: 10.1038/nn1102 (2003). 12937420

[b37] Ostrovsky Y., Meyers E., Ganesh S., Mathur U. & Sinha P. Visual Parsing After Recovery From Blindness. Psychological Science 20, 1484–1491, doi: 10.1111/j.1467-9280.2009.02471.x (2009). 19891751

[b38] Pavlova M., Lutzenberger W., Sokolov A. & N., B. Dissociable cortical processing of recognizable and non-recognizable biological movement: analysing gamma MEG activity. Cereb Cortex 14, 181–188, doi: 10.1093/cercor/bhg117 (2004). 14704215

[b39] Pavlova M., Birbaumer N. & Sokolov A. Attentional modulation of cortical neuromagnetic gamma response to biological movement. Cereb Cortex 16, 321–327, doi: 10.1093/cercor/bhi108 (2006). 15901655

[b40] Händel B., Lutzenberger W., Thier P. & Haarmeier T. Opposite dependencies on visual motion coherence in human area MT+ and early visual cortex. Cereb Cortex 17, 1542–1549, doi: 10.1093/cercor/bhl063 (2007). 16940034

[b41] Saalmann Y. B. & Kastner S. Gain control in the visual thalamus during perception and cognition. Current Opinion in Neurobiology 19, 408–414, doi: 10.1016/j.conb.2009.05.007 (2009). 19556121 PMC3140205

[b42] Sun W. & Dan Y. Layer-specific network oscillation and spatiotemporal receptive field in the visual cortex. Proceedings of the National Academy of Sciences 106, 17986–17991, doi: 10.1073/pnas.0903962106 (2009). PMC2764922 19805197

[b43] Sherman S. M. & Spear P. D. Organization of visual pathways in normal and visually deprived cats. Pshysiological Reviews 62, 738–855 (1982). 10.1152/physrev.1982.62.2.738 6280221

[b44] Vidyasagar T. R. Possible plasticity in the rat superior colliculus. Nature 275, 140–142 (1978). 692682 10.1038/275140a0

[b45] Goldberg M. C., Maurer D., Lewis T. L. & Brent H. P. The Influence of Binocular Visual Deprivation on the Development of Visual-Spatial Attention. Developmental Neuropsychology 19, 53–81, doi: 10.1207/s15326942dn1901_5 (2001). 11411422

[b46] Blatow M. *et al.* A novel network of multipolar bursting interneurons generates theta frequency oscillations in the neocortex. Neuron 38, 805–817 (2003). 12797964 10.1016/s0896-6273(03)00300-3

[b47] Trachtenberg Joshua T. Parvalbumin Interneurons All & Forest No Trees. Neuron 87, 247–248, doi: 10.1016/j.neuron.2015.06.041 (2015). 26182410 PMC6075815

[b48] Trachtenberg J. T. Competition, inhibition, and critical periods of cortical plasticity. Current Opinion in Neurobiology 35, 44–48, doi: 10.1016/j.conb.2015.06.006 (2015). 26126153 PMC5479701

[b49] Troje N. F. & Westhoff C. The inversion effect in biological motion perception: evidence for a “life detector”? Current Biology 16, 821–824, doi: 10.1016/j.cub.2006.03.022 (2006). 16631591

[b50] Troje N. F. Decomposing biological motion: a framework for analysis and synthesis of human gait patterm. Journal of Vision 2, 371–388, doi: 10:1167/2.5.2 (2002). 12678652 10.1167/2.5.2

[b51] Watson A. B. & Pelli D. G. Quest: A bayesian adaptive psychometric method. Perception & Psychophysics 33, 113–120 (1983). 6844102 10.3758/bf03202828

[b52] Anderson A. J. Utility of a dynamic termination criterion in the ZEST adaptive threshold method. Vision Research, 165–171 (2003). 12536138 10.1016/s0042-6989(02)00396-6

[b53] Brainard D. H. The psychophysics toolbox. Spatial Vision 10, 433–437 (1997). 9176952

[b54] Pelli D. G. The videotoolbox software for video psychophysics: transforming numbers into movies. Spatial Vision 10, 437–443 (1997). 9176953

[b55] Tales A., Nweton P., Troscianko T. & Butler S. Mistmatch negativity in the visual modality. NeuroReport 10, 3363–3367 (1999). 10599846 10.1097/00001756-199911080-00020

[b56] Stagg C., Hindley P., Tales A. & S. B. Visual mismatch negativity: the detection of stimulus change. NeuroReport 15, 659–663, doi: 10.1097/01.wnr.0000116966.73984.58 (2004). 15094471

[b57] Comon P. Independent component analysis, a new concept? Signal Processing 36, 287–315 (1994).

[b58] Delorme A. & Makeig S. EEGLAB: an open source toolbox for analysis of single-trial EEG dynamics including independent component analysis. Journal of Neuroscience Methods 134, 9–21, doi: 10.1016/j.jneumeth.2003.10.009 (2004). 15102499

[b59] Oostenveld R., Fries P., Maris E. & Schoffelen J.-M. FieldTrip: open source software for advanced analysis of MEG, EEG, and invasive electrophysiological data. Computational Intelligence and Neuroscience 2011, 1–9, doi: 10.1155/2011/156869 (2011). 21253357 PMC3021840

[b60] Tallon-Baudry C. & Bertrand O. Oscillatory gamma activity in humans and its role in object representation. Trends Cogn Sci 3, 151–163 (1999). 10322469 10.1016/s1364-6613(99)01299-1

