# Supplementary material for: Sight restoration after congenital blindness does not reinstate alpha oscillatory activity in humans
Source: Sci Rep. 2016 Apr 15;6:24683. doi: 10.1038/srep24683 (PMC4832338; doi:10.1038/srep24683)
Supplement: Supplementary Information [file srep24683-s1.pdf]

Title: Sight restoration after congenital blindness does not reinstate alpha oscillatory activity in humans

Authors: *Davide Bottari, Nikolaus F. Troje, Pia Ley, Marlene Hense, Ramesh Kekunnaya and Brigitte Röder

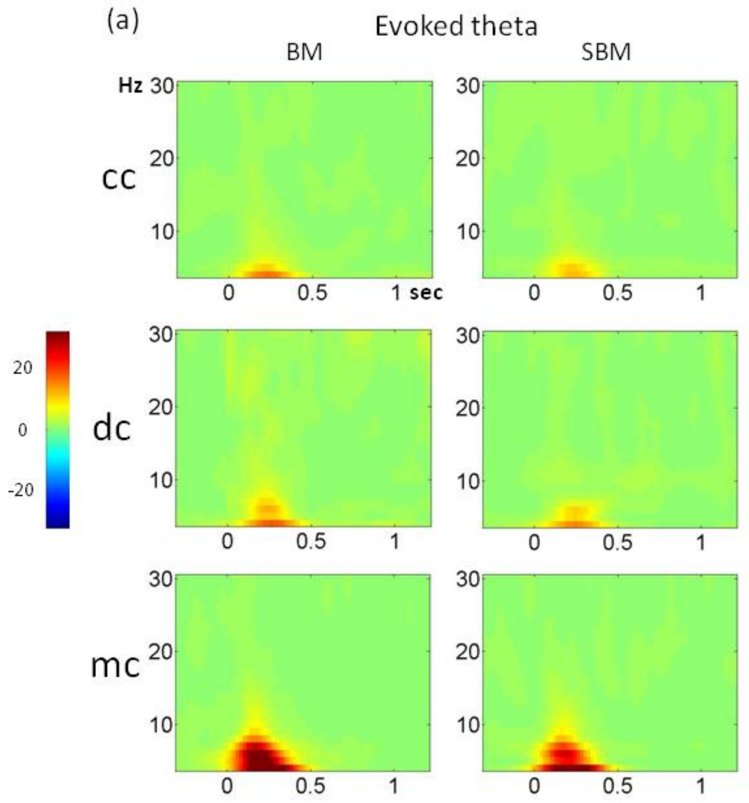

Supplementary Figure 1. (a) *Time frequency representation (relative power signal change compared to baseline) of the evoked oscillatory response between 4 and 30 Hz, averaged across posterior electrodes (TP8/9, P7/8, and O1/2), plotted separately for each group and condition (BM, SBM).*
